# Behaviour of Silica and Florisil as Solid Supports in the Removal Process of As(V) from Aqueous Solutions

**DOI:** 10.1155/2015/562780

**Published:** 2015-03-04

**Authors:** Andreea Gabor, Corneliu Mircea Davidescu, Adina Negrea, Mihaela Ciopec, Lavinia Lupa

**Affiliations:** Faculty of Industrial Chemistry and Environmental Engineering, University Politehnica of Timișoara, Boulevard Vasile Pârvan No. 6, 300223 Timișoara, Romania

## Abstract

In this study two solid supports, silica and florisil, were impregnated with crown ether (dibenzo-18-crown-6) and Fe(III) ions and their efficiency was compared in the adsorption process of As(V) from aqueous solutions. The solid supports were impregnated with crown ether due to their ability to build complexes with positives ions. Fe(III) was used because of As(V) affinity for it. The impregnated solid supports were characterized by energy dispersive X-ray analysis, scanning electron microscopy, Fourier transform infrared spectroscopy, and the specific surface area. The influence of the solid : liquid ratio on the adsorption process, kinetic studies for the pseudo-first-order and pseudo-second-order, and activation energy were studied. Thermodynamic studies as well as equilibrium studies were carried out. The obtained results showed that, from the two considered materials, impregnated silica presents a higher efficiency with a good selectivity, able to remove As(V) from aqueous solutions containing trace concentrations.

## 1. Introduction

It is known that groundwater contains different contaminants. Independently, if the source is natural or anthropogenic, the contaminants have to be eliminated because they represent a threat for human health. One of these contaminants that are worldwide spread is arsenic [1, 2]. In groundwater, arsenic often appears as inorganic form such as arsenate As(V) and arsenite As(III) [3–5]. Because of its toxicity, the World Health Organization (WHO) changed the guidelines from 0.05 to 0.01 mg/L in 1993 and the United States Environmental Protection Agency from 50 *μ*g/L to 10 *μ*g/L in 2002 [3, 4]. Therefore, scientists have the challenge to find different methods to eliminate arsenic from water sources. One of the most efficient technologies to eliminate arsenic from aqueous solutions is the adsorption. The adsorbent used in the adsorption has a big influence on the effectiveness of the process. Silica based adsorbents are known as a good support because they are stabile in acidic conditions and have a high surface area, fast kinetics in the adsorption process, high thermal resistance, and high mass exchange characteristics [6]. Silica particles have been studied worldwide because they have a good adsorption capacity. They can be modified for specific contaminant or species so they become selective and stabile adsorbent [4]. Organized mesoporous silica can be used as a support matrix. Metal oxide can be added onto silica and used for removing a specific pollutant and in catalytic reactions. Organized mesoporous silica has a narrow pore size distribution range and high porosity. Mean pore size, surface area, and porosity can be controlled to a certain extent [7]. Due to their advantages in this paper, a comparative study was made between the adsorbent performance of silica and florisil in the removal process of As(V) from aqueous solutions. In order to enhance the adsorbent properties of these two solid supports, they were impregnated with crown ether and iron ions. For the impregnation of the extractant onto a solid support, four methods were developed: the dry method (DM), wet method, modifier addition method, and dynamic column method (CM) [8]. Crown ether (dibenzo-18-crown-6) was used due to its ability to form complexes with positive ions. Scientists have used this feature and modified the structure of crown ether to profit from this characteristic. Depending on the size of the ion and on the size of its cavity, crown ethers can bind different ions [9–11]. For this reason, it was also loaded with iron ions. Many iron-based adsorbents were developed in the last years [12–15]. Even iron-silica composite was developed [15, 16]. In many cases these adsorbent materials reported in the literature that data developed higher adsorption capacities in the removal process of As(V) from aqueous solutions, but unfortunately they are used to remove arsenic from aqueous solution containing concentration like ppm and are not able to absorb arsenic from underground water containing trace concentration (<100 *μ*g/L), the most often situation found in nature [12–14, 17]. It is useful to encapsulate iron within crown ether that confers favorable hydraulic properties, durability, and mechanical strength. As(V) and As(III) can be removed by iron-based adsorbents from aqueous solutions [3, 18].

In this way, the arsenic selectivity of metal oxides is combined with the durability of a material support [19]. Impregnating the materials with specific affinity positive ions for As and adding specific surface groups for chemisorption can optimize the material removal capacity [7, 20].

## 2. Materials and Methods

### 2.1. Impregnation of the Solid Supports

Silica and florisil were impregnated with crown ether (dibenzo-18-crown-6) and Fe(III) ions using the dry method. Over 1 g of solid support was added to 0,01 g of crown ether (Merck, Germany) used as solvent 25 mL of acetone (VWR Prolabo Chemicals, France). In these solutions, Fe(III) ions were also added in order to obtain a concentration of 400 mg/L from a standard solution of 1 g/L Fe(NO_3_)_3_ (Merck, Germany) in 0,5 mol/L HNO_3_ solution. The sample was kept in contact for 24 h. After that, it was dried at 323.15 K for 24 h.

### 2.2. Characterization of the Obtained Adsorbents

The obtained solid support was characterized by energy dispersive X-ray analysis (EDX) and scanning electron microscopy (SEM), in order to highlight that the impregnation with Fe^III^ occurred, using a scanning electron microscopy (SEM) Quanta FEG 250, equipped with energy dispersive X-ray quantifier (EDAX ZAF). For measuring the specific surface area BET Nova 1200 E Quantachrome was used at a temperature of 77 K with nitrogen (N_2_). The samples were degassed with vacuum for 5 hours at room temperature. The impregnation of the solid supports with crown ether and iron ions was also evidenced by FTIR analysis. The FTIR spectra (KBr pellets) of the obtained adsorbent were recorded on a Shimadzu Prestige-21 FTIR spectrophotometer in the range 4000–400 cm^−1^.

### 2.3. Adsorption Experiments

For this experiment, a stock solution of arsenic of 1 g/L As(V) solution (Merck Standard Solutions) was used. Other solutions of As(V) ions were prepared from the stock solution by appropriate dilution. The experiments were performed at an initial pH of 7-8. It was adjusted using NaOH and HNO_3_ solutions of different concentrations. The pH was measured with a Knick pH-Meter 765 Calimatic fitted with a glass electrode which had been calibrated using various buffer solutions. First the variation of solid : liquid ratio (S : L) was studied. 0,1; 0,2; 0,3; 0,4; 0,5 g of impregnated silica and of impregnated florisil were mixed with 25 mL solution of arsenic having a concentration of 100 *μ*g/L, for one hour. A Julabo SW23 mechanical shaker bath was used at 200 rot/min and at a temperature of 298 K. The filtrate was collected for As(V) analysis.

To study the effect of the contact time, 0.1 g of impregnated silica, 0.5 g of impregnated florisil, and 25 mL solution of arsenic were shacked for 30, 60, 120, 180, and 240 minutes. This was carried out for three temperatures: 298, 308, and 318 K. The samples were then filtrated and the residual concentration of As(V) was analyzed.

The effect of the initial As(V) concentration (*C*_0_ = 25, 50, 75, 100, 125, and 150 *μ*g/L) was also studied. The samples were kept in contact for one hour with 25 mL arsenic solution with different concentrations, keeping the same solid : liquid ratio. The filtrate was collected for As(V) analysis. All As(V) analyses were made using inductively coupled plasma mass spectrometry ICP-MS Bruker Aurora M90.

## 3. Results and Discussion

### 3.1. Characterization of Silica and Florisil Impregnated with Crown Ether and Fe(III)

**Figure 1:**
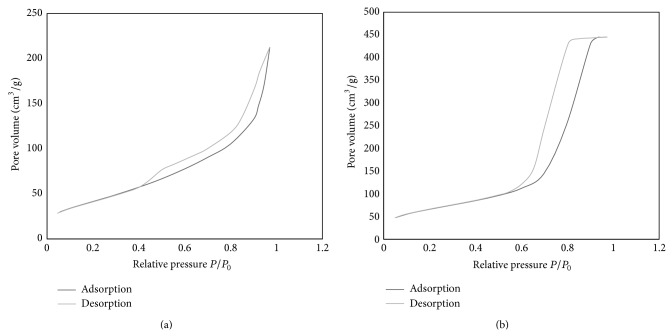
N_2_ adsorption/desorption isotherm of (a) florisil and (b) silica.

From the BET measurements, the specific surface area and total pore volume were determined (Table 1).  Figure 1 presents the N_2_ adsorption and desorption of impregnated florisil and silica. In Figure 1, it can be noted that the silica samples display a higher mesoporous volume than the florisil samples. In case of silica samples, the isotherms are characteristic for isotherms of type IV and a H1 hysteresis loop. Type IV isotherms occur on porous adsorbents possessing pores in the radius range of approximately 15–1000 angstroms (A). The slope increase at higher elevated pressures indicates an increased uptake of adsorbate as the pores are being filled. Type H1 hysteresis loops are typical for adsorbents with well-defined structures and narrow pore size distributions. This type of isotherms presents a hysteresis loop which is specific for mesoporous materials which present a capillary condensation. The increase in the N_2_ adsorbed volume is higher in the region of 0.7 and 0.9 relative pressure. The macropores volume of silica materials is very limited as shown by the plateau achieved for N_2_ adsorption isotherms at relative pressure between 0.9 and 1.0. The samples with florisil present a type IV isotherm and a H3 hysteresis loop. Type H3 loops are usually given by aggregates of plate-like particles or adsorbents containing slit-shaped pores [7, 21–25].

Table 1 summarizes the physical characteristics of the materials after impregnation and it can be noticed that the impregnated silica presents a higher surface area and porosity than the florisil sample. For this reason, it is expected that the impregnated silica will develop a higher adsorption capacity in the removal process of As(V) from aqueous solutions.

**Figure 2:**
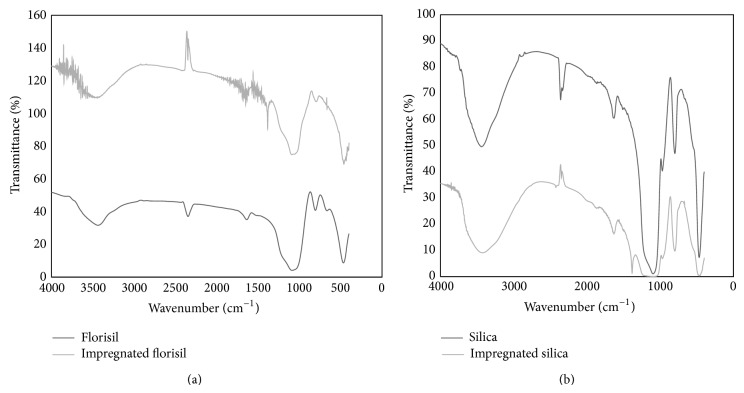
IR vibrational spectrum of studied adsorbents material. (a) Florisil. (b) Silica.

The florisil and silica impregnated with crown ether and Fe(III) ions were subjected to the FTIR analysis in order to prove that the impregnation occurred. The FTIR spectrum is provided in Figure 2. The intense bands around 1100 cm^−1^ are attributed to the *ν*_sym_ (C_aliphatic_-O-C_aromatic_) and *ν*_sym_ (C_aliphatic_-O-C_aliphatic_), respectively, which prove that the impregnation with dibenzo-18-crown-6 crown ether occurred [26, 27]. In this interval, the adsorption bands are overlapped (between 1000 and 1200 cm^−1^) which are due to antisymmetric stretching mode and symmetric stretching mode of Si-O-Si, respectively [11]. It is more obvious in case of the silica adsorbent. Figure 2 also has shown that the bending vibration bands of CH_2_ groups are presented at about 1460 cm^−1^ [28]. The FTIR spectrum shows a wider and higher peak at 3430 cm^−1^ in case of silica sample compared to florisil, revealing a higher content of O-H bond, which come from a higher presence of Fe-OH groups [7]. The IR spectrum confirms the fact that both studied solid supports were impregnated with crown ether and Fe(III) ions. In case of silica, the impregnation process obviously suggests a higher quantity of crown ether and Fe(III) ions than in case of florisil. Therefore, the FTIR analysis suggests also that the impregnated silica is possible to develop a higher adsorption capacity.

**Figure 3:**
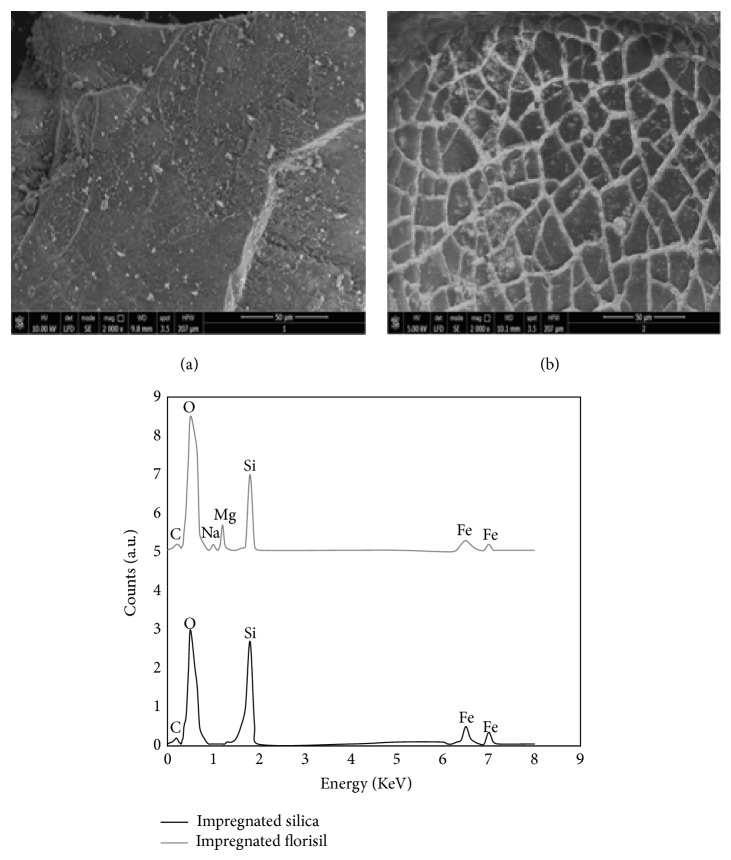
SEM images and EDX spectrum of impregnated florisil (a) and impregnated silica (b).

The surface morphology and the EDX quantification of the obtained material are presented in Figure 3. The spherical shape of the micron sized silica particle presents a higher aggregation following surface treatment. Also it can be observed that the coating process with Fe(III) ions is inside the pore in case of silica rather than homogenous coating like in the case of florisil. The EDX quantification proved that the studied solid supports were impregnated with crown ether and loaded with Fe(III) ions.

### 3.2. As(V) Adsorption

#### 3.2.1. Influence of the S : L Ratio on the Adsorption of As(V)

**Figure 4:**
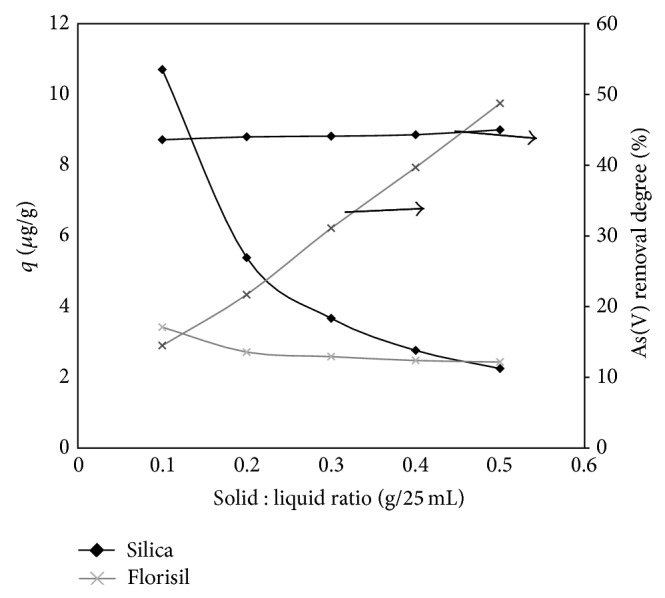
Influence of the S : L ratio on the removal of As(V) from aqueous solutions for impregnated silica and florisil.

Figure 4 presents the dependence of the adsorption capacity on the removal of As(V) from aqueous solutions for impregnated silica and florisil versus the S : L ratio.

Increasing the amount of impregnated silica used for removing As(V) from aqueous solutions leads to decreasing of the adsorption capacity because the adsorption capacity is in relation to the amount of adsorbent used. In the same time, the removal degree of As(V) from aqueous solution is not influenced by the amount of impregnated silica used in the adsorption process. In contrast to silica, the adsorption capacity for florisil remains almost constant for the S : L ratio used, but the As(V) removal degree increases with the increasing of the S : L ratio. In order to obtain both higher adsorption capacity and higher removal degree, the optimal S : L ratio used for future experiments is 0.1 g impregnated silica and 0.5 g impregnated florisil for 25 mL of As(V) aqueous solutions.

#### 3.2.2. Kinetic Studies

**Figure 5:**
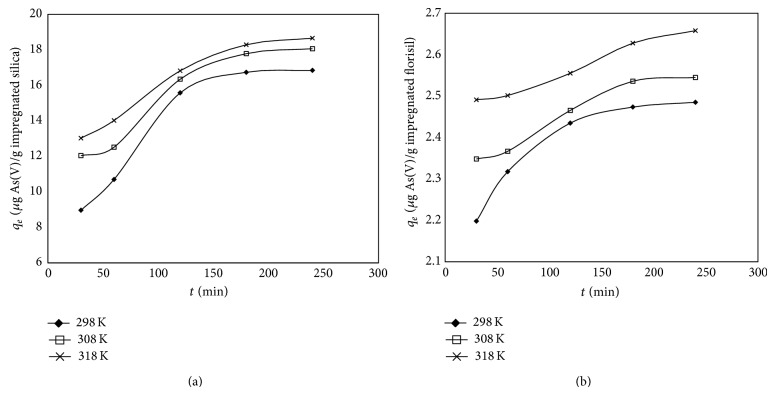
Influence of the contact time on the adsorption capacity of the impregnated silica (a) and on the impregnated florisil (b).

For the kinetic studies, the influences of the contact time on the adsorption capacity of the impregnated silica and florisil were studied (Figure 5). The data showed that the adsorption capacity for both materials increases with increase of time till they reach the equilibrium at 180 minutes. The increase of the adsorption capacity with the time increasing in case of silica is very obvious; in case of florisil, this increase is not significant. The adsorption capacity of both of the studied materials increases with the temperature increasing indicating an endothermic nature of As(V) adsorption process.

**Figure 6:**
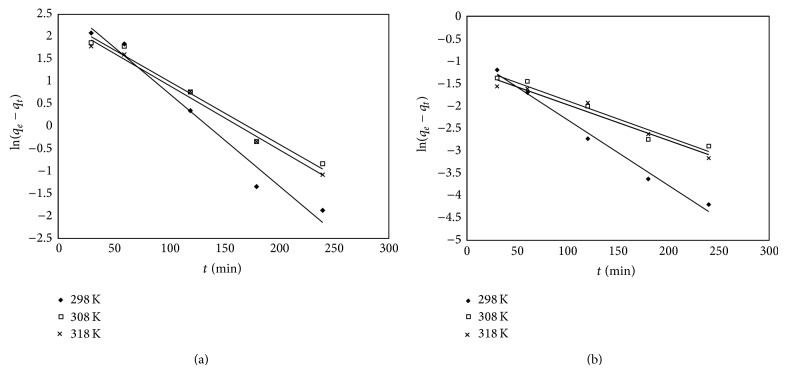
Pseudo-first-order kinetic plots for silica (a) and florisil (b) at the adsorption of As(V).

**Figure 7:**
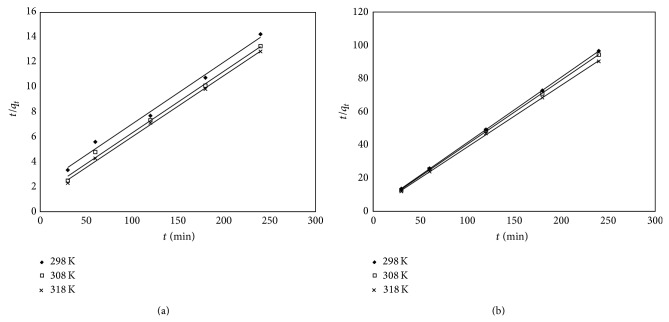
Pseudo-second-order kinetic plots for silica (a) and florisil (b) at the adsorption of As(V).

The experimental data were fitted with the pseudo-first-order (Figure 6) and the pseudo-second-order (Figure 7) kinetic models. The following equation defines the two kinetic models:

(1) ln⁡⁡qe−qtln⁡⁡qt−k1t,tqt=1k2qe2+tqe,

where *q*_*e*_ and *q*_*t*_ are the amount of adsorbate onto the adsorbent (*μ*g/g) at equilibrium and at time *t*, respectively, *t* is the contact time (min), *k*_1_ is the pseudo-first-order adsorption rate constant (min^−1^), and *k*_2_ is the pseudo-second-order adsorption rate constant (g/*μ*g·min). The rate constant *k*_1_ and the correlation coefficients were calculated from the slope and intercept of the linear representation of ln(*q*_*e*_ − *q*_*t*_) versus *t* (Figure 6) [29]. The rate constant *k*_2_, the *q*_calc_, and the corresponding linear regression coefficients were calculated from the linear plots of *t*/*q* against *t* (Figure 7) [30]. The values of constants, together with the regression coefficients (*R* ^2^) obtained in all the cases, are summarized in Table 2.

The low correlation coefficients obtained for the pseudo-first-order kinetic model for impregnated silica and florisil as well as the difference between the experimental and the calculated adsorption coefficient show that it is not appropriate to fit the experimental data with this kinetic model. The values obtained for the pseudo-second-order kinetic model fit better the experimental data and represent the adsorption process of As(V) onto impregnated silica and florisil. Comparing the values for silica and florisil, it can be concluded that the adsorption process of As(V) on silica went much better than that on florisil.

**Figure 8:**
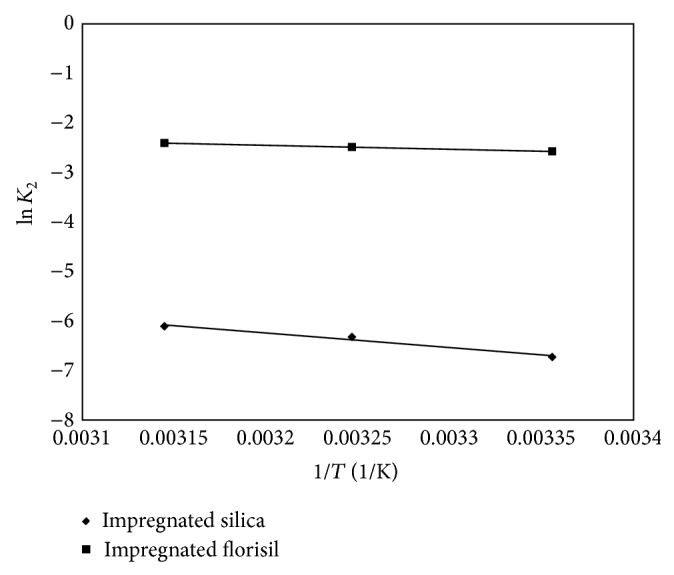
Arrhenius plot of As(V) adsorption onto impregnated silica and florisil.

To calculate the activation energy of the adsorption of As(V) on impregnated silica and florisil, the Arrhenius equation was used with the rate constant from the pseudo-second-order kinetic model:

(2) $$\begin{array}{c} \ln k_{2}=\ln A-\frac{E}{RT}, \end{array}$$

where *k*_2_ is the pseudo-second-order rate constant of sorption (g/min·*μ*g), *A* is the Arrhenius constant which is a temperature independent factor (min·g/*μ*g), *E* is the activation energy of sorption (kJ/mol), and *T* is the absolute temperature (K). The activation energies were calculated from the slope of the plots of ln*k*_2_ versus 1/*T* (Figure 8).

The activation energy calculated was 24.77 kJ/mol for impregnated silica and 1.118 kJ/mol for impregnated florisil. These values suggest that the adsorption of As(V) onto impregnated silica is a chemical sorption in comparison to the adsorption process onto impregnated florisil that is a physisorption. These conclusions suggest also that the impregnated silica could develop a higher maximum adsorption capacity than the impregnated florisil in the removal process of As(V) from aqueous solutions.

#### 3.2.3. Thermodynamic Studies

The thermodynamic studies were made to illustrate whether the adsorption process is a spontaneous process or not. Therefore, the equilibrium constant *K*_*c*_ is defined as follows [31]:

(3) $$\begin{array}{c} K_{c}=\frac{C_{A}}{C_{c}}, \end{array}$$

where *C*_*A*_ is the solid phase concentration at equilibrium (*μ*g/L), *C*_*c*_ is the equilibrium concentration (*μ*g/L), *T* (K) is the absolute temperature, and *R* is the gas constant.

If the equilibrium constant changing with the temperature is used, thermodynamic parameter such as the free energy change Δ*G*°, enthalpy change Δ*H*°, and entropy change Δ*S*° can be estimated by

(4) $$\begin{array}{c} \Delta G^{{}^{\circ}}=-RT\ln K_{c}, \end{array}$$

where *K*_*c*_ is the equilibrium constant, *T* (K) is the absolute temperature, and *R* is the gas constant.

The relations between the thermodynamic parameters are as follows:

(5) ΔG°ΔH°−TΔS°ln⁡⁡Kc=ΔS°R−ΔH°RT.

**Figure 9:**
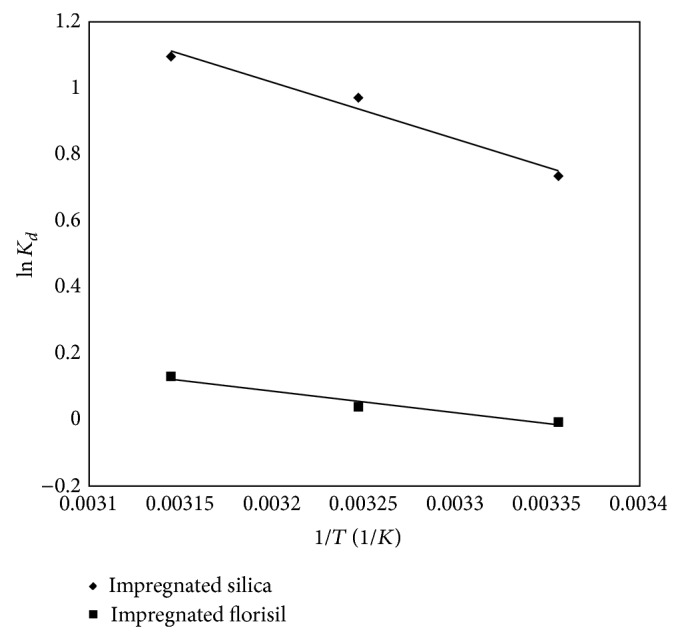
Temperature effect on the adsorption of As(V) onto impregnated silica and florisil.

From the plot in ln⁡⁡*K*_*c*_ versus 1/*T* (Figure 9), the values of Δ*S*° can be determinate which are summarized in Table 3.

All Δ*G*° values are negative for silica which means that the adsorption process of As(V) is a spontaneous process. In case of impregnated florisil, the adsorption process is spontaneous only at higher temperature. For both studied materials, Δ*G*° decreases with increase of temperature. The adsorption of As(V) on the materials is an endothermic process due to the positive Δ*H*° values obtained. The positive values of Δ*S*° indicate an increased randomness at the solid-solution interface during the adsorption process [32].

#### 3.2.4. Equilibrium Studies

**Figure 10:**
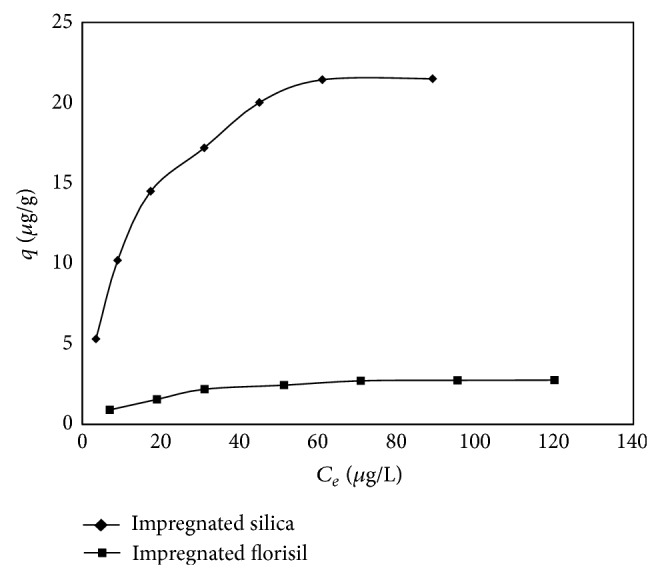
Adsorption isotherm of As(V) onto impregnated silica and florisil.

The adsorption process of As(V) removal by impregnated silica and florisil is presented in Figure 10.

The adsorption capacity increases with the increase of the equilibrium concentration of As(V) onto the impregnated materials till it approaches a constant value at the highest equilibrium concentration. It can be noticed that the impregnated silica develops much higher adsorption capacity in the removal process of As(V) from aqueous solutions than the impregnated florisil. These results are in accordance with the conclusions raised from the characterization of the obtained materials and the conclusion raised from the activation energy.

To describe the equilibrium nature of the As(V) adsorption process onto impregnated silica and florisil, the Langmuir and Freundlich isotherm models were used.

**Figure 11:**
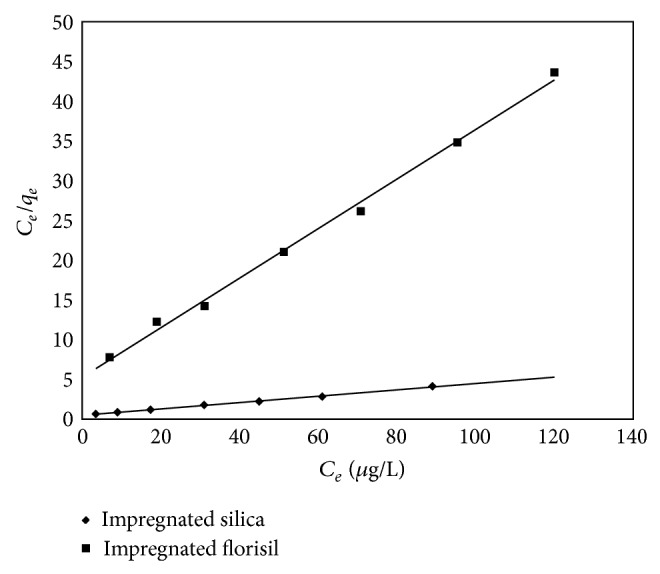
Langmuir isotherm for As(V) adsorption on impregnated silica and florisil.

The Langmuir isotherm can be expressed in linear form

(6) $$\begin{array}{c} \frac{C_{e}}{q_{e}}=\frac{1}{K_{L}q_{m}}+\frac{C_{e}}{q_{m}}, \end{array}$$

where *q*_*e*_ is the amount of As(V) adsorbed per gram adsorbent, *q*_*m*_ is the monomolecular adsorption capacity (*μ*g/g), *C*_*e*_ is the equilibrium concentration of the adsorbate in solution after the adsorption (*μ*g/L), and *K*_*L*_ is the Langmuir constant. From the linear plot of *C*_*e*_/*q*_*e*_ versus *C*_*e*_, the values for *K*_*L*_ and *q*_*m*_ can be calculated (Figure 11) [8].

**Figure 12:**
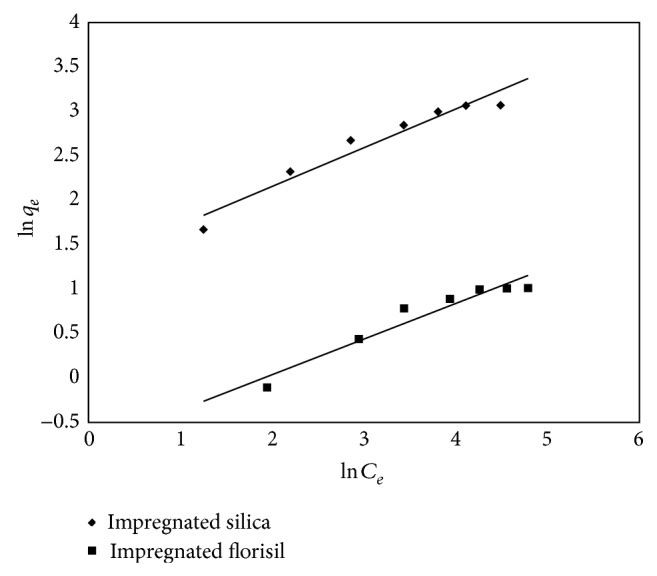
Freundlich isotherm for As(V) adsorption on impregnated silica and florisil.

The Freundlich isotherm can be expressed in linear form by

(7) $$\begin{array}{c} \ln q_{e}=\ln K_{F}+\frac{1}{n}\ln C_{e}, \end{array}$$

where *q*_*e*_ is the amount of As(V) adsorbed per gram adsorbent (*μ*g/g), *C*_*e*_ is the equilibrium concentration of the adsorbate in solution after the adsorption (*μ*g/L), and *K*_*F*_ is the Freundlich constant. 1/*n* and *K*_*F*_ can be determined by plotting ln⁡⁡*q*_*e*_ versus ln⁡⁡*C*_*e*_ (Figure 12).

Table 4 presents the parameters of Langmuir and Freundlich isotherms for the adsorption of As(V) onto impregnated silica and florisil.

Comparing the regression coefficient of the two isotherms, the Freundlich isotherm has a lower value than the Langmuir isotherm for both used materials. This suggests that the Langmuir model was more favorable, including the fact that a low difference between the *q*_*m*,exp⁡_ and *q*_*m*,calc_ was observed. The Langmuir model describes an adsorption over a homogeneous surface and that all adsorption sites are energetically equivalent and do not affect the adsorption of molecules on an adjacent site [6, 33].

## 4. Conclusions

The present study showed that the adsorption efficiency of silica and florisil in the removal process of As(V) from aqueous solutions was increased by impregnation of the solid support with crown ether and loaded with Fe(III) ions. The obtained materials were characterized through SEM, EDX, BET, and FTIR analysis. The kinetic studies revealed that the adsorption process of As(V) onto the studied materials followed the pseudo-second-order kinetic model. The equilibrium data were fitted with Langmuir and Freundlich isotherm, the best correlation being obtained by the Langmuir one. The characterization analysis together with As(V) adsorption tests suggested that the impregnation process in case of silica solid support was more obvious than in case of florisil which leads to an excellent adsorption performance of As(V) when the silica solid support was used. The use of silica impregnated with crown ether and Fe(III) as adsorbent, in the removal process of As(V) from aqoueous solutions, represents an efficient method, because a good efficiency and selectivity of As(V) removal from aqueous solutions containing trace concentration is obtained. Also is achieved a residual concentration of As(V) under the maximum level admitted by the World Health Organization (<10 ppb).

## Figures and Tables

**Table 1:** BET analysis summary of impregnated silica and florisil.

	Surface area (m^2^/g)	Pore volume (cc/g)	Pore width (nm)
Impregnated silica	237	0.663	5.69
Impregnated florisil	155	0.274	3.63

**Table 2:** Kinetic parameters for As(V) adsorption onto studied material.

Temperature (K)	*q*_exp⁡_ (*µ*g/g)	Pseudo-first-order			Pseudo-second-order		
		*k*_1_ (1/min)	*q*_calc_ (*µ*g/g)	*R* ^2^	*k*_2_ (g/*µ*g·min)	*q*_calc_ (*µ*g/g)	*R* ^2^
Impregnated silica							
298	17	0.0207	16.66	0.9730	1.195 · 10^−3^	20.12	0.9919
308	18.5	0.0141	11.25	0.9781	1.791 · 10^−3^	20.20	0.9950
318	19	0.0143	10.56	0.9895	2.225 · 10^−3^	20.32	0.9978

Impregnated florisil							
298	2.5	0.0146	0.42	0.9887	0.077	2.53	0.9999
308	2.6	0.0081	0.33	0.9614	0.083	2.59	0.9998
318	2.7	0.0079	0.30	0.9634	0.090	2.69	0.9997

**Table 3:** Thermodynamic parameters.

	Δ*H*° (kJ/mol)	Δ*S*° (J/mol·K)	Δ*G*° (kJ/mol)			*R* ^2^
			298 K	308 K	318 K	
Silica	14.244	0.054	−1.859	−2.4	−2.940	0.9752
Florisil	5.417	18.05	0.03816	−0.14234	−0.32284	0.9577

**Table 4:** Parameters of Langmuir and Freundlich isotherms for the adsorption of As(V) onto impregnated silica and florisil.

	*q*_*m*,exp⁡_ (*µ*g/g)	Langmuir isotherm			Freundlich isotherm		
		*K*_*L*_ (L/*μ*g)	*q*_*m*,calc_ (*µ*g/g)	*R* ^2^	*K*_*F*_ (*μ*g/g)	1/*n*	*R* ^2^
Silica	21.5	0.078	25	0.9975	3.61	0.4355	0.9427
Florisil	2.75	0.059	3.20	0.9959	0.47	0.4005	0.9270
